# Editorial: Emerging aspects of ketone metabolism in health & disease

**DOI:** 10.3389/fphys.2024.1404454

**Published:** 2024-04-03

**Authors:** Brianna Jane Stubbs, Kenneth M. Ford, Jeff Volek

**Affiliations:** ^1^ Buck Institute for Research on Aging, Novato, CA, United States; ^2^ Florida Institute of Human and Machine Cognition, Pensacola, FL, United States; ^3^ The Ohio State University, Colombus, OH, United States

**Keywords:** ketosis, ketogenic diet, ketone ester, exogenous ketone, ketone infusion, butanediol

In recent years, the metabolic state of ketosis has been the subject of increased interest in the scientific, medical, and lay community. Traditionally viewed with skepticism due to the negative associations of high fat diets, as well as the inappropriate conflation of nutritional/physiological ketosis with pathological ketoacidosis, ketones are now recognized as an impactful signaling metabolite, with a role extending far beyond a simple energy substrate. Applications of ketosis under active investigation are highly diverse, ranging from optimizing athletic performance to mitigating cardiometabolic or neurodegenerative disorders. This Research Topic aimed to highlight emerging aspects of how ketone metabolism could be applied in health and disease.

Ketogenic diets, characterized by high fat, moderate protein, and low carbohydrate intake, represent the most established method for inducing and sustaining ketosis. Given the long history of use, it was perhaps somewhat surprising that only one article in this Research Topic focused on a ketogenic diet intervention. Noakes et al. contributed a review article that combined discussion of a foundational exercise physiology concept, the cross over point, with data from athletes following ketogenic diets (‘fat-adapted’ athletes), which challenges the normal expectations of the cross-over point. They point out the crossover point can occur at a higher exercise intensity than usually predicted in fat-adapted athletes and that far higher absolute rates of fat oxidation can occur in these athletes compared to athletes on a higher carbohydrate diet. Furthermore, they highlight that a non-trivial proportion of middle-aged athletes present with pre-diabetic glycemic values, and suggest that a ketogenic, low carbohydrate, high fat diet could at least maintain physical performance, alongside potentially improved glycemic control.

The remainder of articles included in this Research Topic focus on a non-traditional method of achieving ketosis, the consumption of exogenous ketones. This highlights the rapid growth of interest in exogenous ketones, which is further emphasized by the opinion piece by Todd King, that describes how ‘ambiguity begets uncertainty’ concerning the naming of different types of exogenous ketones in use in research and in the consumer market. Whilst there are multiple classes of exogenous ketones, broadly, ketone salts, ketone precursors and ketone esters, King focusses his discussion on ketone esters; how different molecular structures might result in different functional effects and therefore molecules must be correctly identified to facilitate interpretation of any results. King ultimately cautions against the use of ‘ketone esters’ as a generic term, suggesting that a consensus on naming of these molecules might facilitate progress in their application. This opinion piece highlights a topic that is of increasing importance with the growing commercial availability and widespread use of exogenous ketone compounds.

Exogenous ketones are putative tools to deliver the benefits of ketosis without other dietary manipulation. Rushing et al. describe the use of an acetoacetate ketone di-ester in a preclinical model of metabolic disease. They found that the ketone ester attenuated the accretion of adiposity and reduced markers of liver steatosis, inflammation, ballooning, and fibrosis in lean mice placed on a high fat, high sugar diet. Their results demonstrate that exogenous ketones may still be efficacious even in the absence of carbohydrate restriction, implicating a direct effect of ketone metabolism or signaling effects.

The remaining three articles in the Research Topic address general dosing, kinetic and safety characteristics of three different types of exogenous ketones in humans: ketone infusions, fatty acid ketone di-esters, and the ketone precursors, (R)-1,3-butanediol. Storoschuk et al. undertook a systematic review and meta-analysis to determine the relationship between intravenous ketone infusion rate and blood ketone concentration, notably accounting for the delivery of racemic ketone salts and the use of non-enantiomer specific measurement methods. This work provides a foundation for designing dosing strategies for exogenous ketones, where there is a target therapeutic blood concentration. Both Mah et al. and Lowder et al. report on acute ketone kinetic effects and safety/tolerance of different exogenous ketone beverages. Mah et al. describe the ketone and ketone ester metabolite kinetics of escalating doses of the fatty acid ketone di-ester bis hexanoyl (R)-1,3 butanediol before and after a 1-week period of daily dosing. They found that target analytes increased with serving size but were not changed after 1 week of ingestion, suggesting no metabolic adaptation occurred during this time. Lowder et al. describe the acute blood ketone response to three drinks containing the ketogenic alcohol, (R)-1,3 butanediol taken over several hours. They found these ketone drinks were well tolerated and induced nutritional ketosis. Together, these articles provide context for exogenous ketone dosing and compound selection for future studies.

In conclusion, the growing body of evidence surrounding translational applications of endogenous and exogenous ketosis underscores its potential as a powerful tool for promoting health and combating disease. Currently there are 132 trials using exogenous ketones, and 395 trials using ketogenic diet listed on clinicaltrials.gov. This increase in research interest will go some way towards elucidating the mechanisms underlying the functional effects of ketosis and hopefully provide evidence to inform the use of ketogenic strategies to improve human health and performance.

